# Metabolomics of the Bio-Degradation Process of Aflatoxin B1 by Actinomycetes at an Initial pH of 6.0

**DOI:** 10.3390/toxins7020439

**Published:** 2015-02-04

**Authors:** Manal Eshelli, Linda Harvey, RuAngelie Edrada-Ebel, Brian McNeil

**Affiliations:** 1 Food Science and Technology Department, Faculty of Agriculture, University of Tripoli, Tripoli, Libya; 2 Fermentation Laboratory, Strathclyde Institute of Pharmacy and Biomedical Sciences, University of Strathclyde, 161 Cathedral Street, Glasgow G4 0RE, UK; E-Mails: L.M.Harvey@strath.ac.uk (L.H.); ruangelie.edrada-ebel@strath.ac.uk (R.E.-E.)

**Keywords:** aflatoxin B_1_, biodegradation, microorganisms, metabolites, high-pressure liquid chromatography tandem mass spectrometry (LC-MS/MS)

## Abstract

Contamination of food and feed by Aflatoxin B1 (AFB1) is a cause of serious economic and health problems. Different processes have been used to degrade AFB1. In this study, biological degradation of AFB1 was carried out using three Actinomycete species, *Rhodococcus erythropolis* ATCC 4277, *Streptomyces lividans* TK 24, and *S. aureofaciens* ATCC 10762, in liquid cultures. Biodegradation of AFB1 was optimised under a range of temperatures from 25 to 40 °C and pH values of 4.0 to 8.0. An initial concentration of 20 µg/mL of AFB1 was used in this study. The amount of AFB1 remaining was measured against time by thin layer chromatography (TLC) and high-performance liquid chromatography (HPLC), coupled with UV and mass spectrometry (LC-MS). All species were able to degrade the AFB1, and no significant difference was found between them. AFB1 remained in the liquid culture for *R. erythropolis*, *S. lividans* and *S. aureofaciens* were 0.81 µg/mL, 2.41 µg/mL and 2.78 µg/mL respectively, at the end of the first 24 h. Degradation occurred at all incubation temperatures and the pH with the optimal conditions for *R. erythropolis* was achieved at 30 °C and pH 6, whereas for *S. lividans* and *S. aureofaciens* the optimum conditions for degradation were 30 °C and pH 5. Analysis of the degradative route indicated that each microorganism has a different way of degrading AFB1. The metabolites produced by *R. erythropolis* were significantly different from the other two microorganisms. Products of degradation were identified through metabolomic studies by utilizing high-resolution mass spectral data. Mass spectrometric analysis indicated that the degradation of AFB1 was associated with the appearance of a range of lower molecular weight compounds. The pathway of degradation or chemical alteration of AFB1 was followed by means of high resolution Fourier transform mass spectrometry (HR-FTMS) analysis as well as through the MS^2^ fragmentation to unravel the degradative pathway for AFB1. AFB1 bio-degradation was coupled with the accumulation of intermediates of fatty acid metabolism and glycolysis. A plausible mechanism of degradation of AFB1 by *Rhodococcus* was hypothesized.

## 1. Introduction

**Figure 1:**
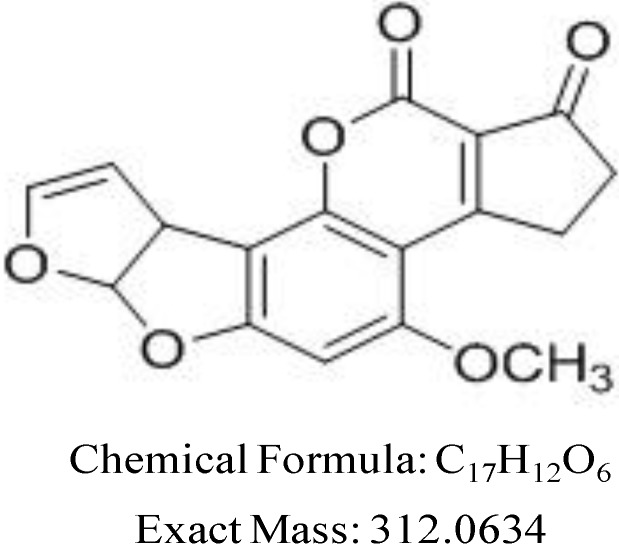
Chemical structure for AFB1.

Mycotoxin can contaminate food and feed as a result of the growth of fungi before harvesting or because of improper storage [1]. It can be found in almost 25% of the world’s agricultural commodities [2]. Aflatoxins are mycotoxin secondary metabolites and are the most hazardous mycotoxins. Aflatoxins were first isolated and characterized following the death of more than 100,000 turkeys from an unidentified disease. The toxin was traced to consumption of a mould-contaminated peanut meal [1]. Among eighteen different types of aflatoxins identified, the major members are aflatoxin B_1_, B_2_, G_1_ and G_2_. AFB1 is the most toxic and usually prevalent in cultures as well as in food products [3]. AFB1 is a difuranocoumarin derivative (Figure 1) and is considered to be heat stable when molecules are within the range of conventional food processing temperatures (80–121 °C) [4]. Once the food is contaminated with aflatoxins, there are only two options: either the toxin is removed, or the toxin is degraded into less toxic or non-toxic compounds [5].

Different physical and chemical methods have been developed and tested to control AFB1. Disadvantages of these methods are limited in their practical applications due to nutritional losses, sensory quality reduction, and high cost. Ideally, any detoxification procedure should consider reducing the concentration of toxins to safe levels, preventing production of toxic degradation products and avoiding the loss of nutritional value of treated products [6,7,8,9]. The application of enzymes or microorganisms is an alternative method to detoxify AFB1 in contaminated food and feed [8,10]. A number of fungal species such as *Pleurotus ostreatus* [11], *Rhizopus* sp. [6,12], *Armillariella tabescens* [6], and *Trichoderma* strains, [13] as well as the yeast *Saccharomyces cerevisiae* [14] have been found to be able to transform AFB1 into less toxic metabolites. However, the use of fungi and yeasts is not economical because of the extraction process and lengthy incubation time. Reports recorded the reduction of AFB1 by lactic acid bacteria such as *Lactobacillus* sp. [15], *Lactococcus* sp. [5,16], and *Bifidobacterium* sp. [17]. The reduction of AFB1 by lactic acid bacteria was due to AFB1 binding to bacterial cell walls [18]. Therefore, complete toxin removal from contaminated food or feed is difficult or impossible [5,18].

Soil microorganisms were also examined for their ability to degrade AFB1 [15]. The effective bacteria were limited to Actinomycetes bacteria, including *R.* *erythropolis*, *Mycobacterium fluoranthenivorans*, *Flavobacterium aurantiacum* and *Stenotrophomonas maltophilia* [19,20,21]. The efficiency of *R. erythropolis* culture on AFB1 degradation was first reported in 2006. *Rhodococcus* are anaerobic, Gram-positive bacterium capable of transforming a wide range of xenobiotic compounds [22]. *Rhodococcus* has a large genome, and mega-plasmids make them suitable industrially for biotransformation and biodegradation [10]. In addition, *Streptomyces* also has a long history in the industry of antibiotic production and can metabolize aromatic compounds by producing extracellular enzymes [23,24]. This seems to be a potentially promising prospect for the biodegradation of AFB1 [25,26]. To establish bio-decontamination methods, greater understanding of AFB1 metabolism by these microorganisms is required, particularly in terms of optimizing the biodegradation conditions, pathways, and the function of the respective enzymes involved in the degradation. Therefore, the focus in this study was to investigate the degradation of AFB1 by Actinomycetes liquid cultures and determine the optimum conditions for AFB1 degradation that involves the variable factors: pH, temperature, and incubation time. Additionally, the aim was to identify the degradation products by using the tools of metabolomics, which involved high resolution FTMS (Fourier Transform Mass Spectrometry) and an automated label-free differential expression analysis software (SIEVE).

## 2. Results and Discussion

### 2.1. AFB1 Degradation by Actinomyces in Liquid Culture

**Figure 2:**
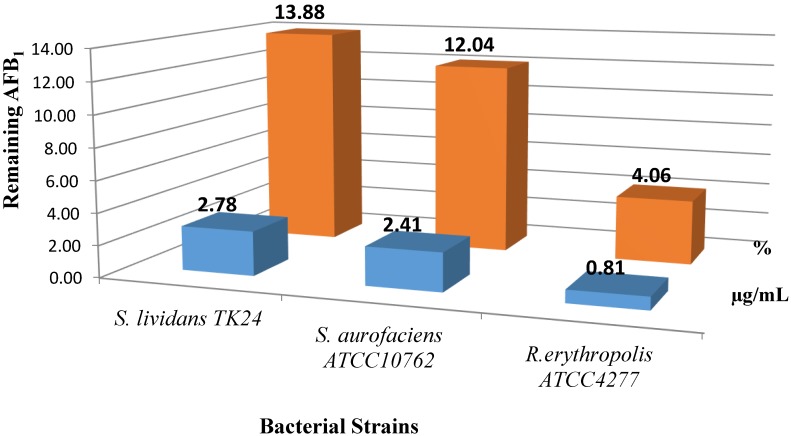
AFB1 degradation by cultures *of R. erythropolis ATCC 4277*, *S. lividans TK 24* and *S. aureofaciens* ATCC10762 after 24 h incubation at 30 °C and pH 6.0.

Liquid cultures of *R. erythropolis* ATTC 4277, *S. lividans TK 24* and *S. aureofaciens* ATCC10762 were exposed to 20 µg/mL of AFB1 for 24, 48, and 72 h. All strains were able to degrade AFB1 in ISP medium No. 1. As illustrated in Figure 2, a significant reduction of AFB1 to 0.81 µg/mL was observed after 24 h incubation. This meant that only 4% of AFB1 remained in the culture with *R. erythropolis* ATCC 4277 at 30 °C and pH 6.0. Meanwhile, the remaining amount of AFB1 was slightly higher when *Streptomyces* strains were used under the same conditions: 12% (*S. lividans*) and 14% (*S. aureofaciens*) of the initial concentration after 24 h incubation. The control sample (AFB1 + media) was stable over the period degradation in ISP No. 1 media. This study complements the range of soil bacteria studied which has the ability to degrade AFB1 *in vitro* during incubation. Evidence was revealed in this research for AFB1 degradation by these microorganisms. This was in agreement with Ciegler *et al.*, (1966) [25] who reported that *Flavobacterium aurantiacum* NRRL B-184, showed a high capacity for degrading of AFB1. It was considered to be the only bacteria able to degrade AFB1 [25]. However, recently, other soil microbes have also been tested for degrading AFB1 [19,26]. The liquid cultures and cell-free extract of *R. erythropolis* DSM 14303, *Nocardia corynebacterioides* DSM 12676, *N. corynebacterioides* DSM 20151, and *Mycobacterium fluoranthenivorans* sp. nov. DSM 44556T degraded AFB1 effectively [19].

### 2.2. Optimising AFB1 Degradation Conditions

**Figure 3:**
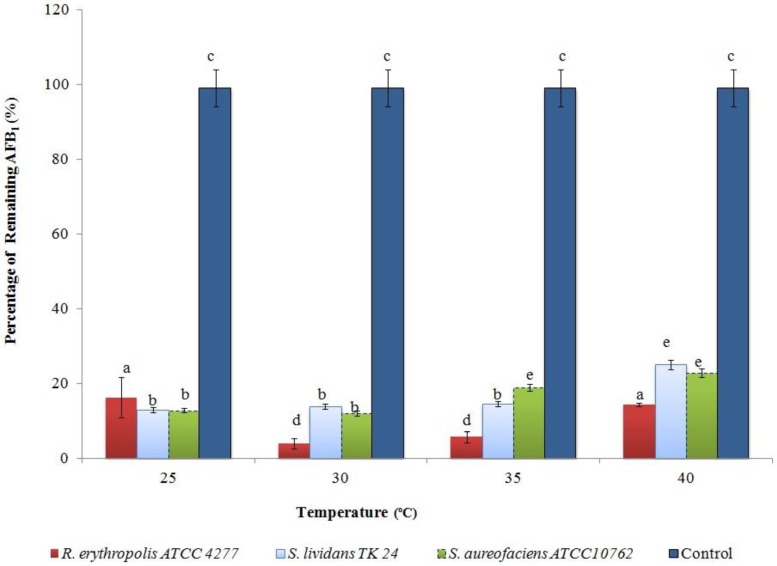
The effect of incubation temperature on AFB1 degradation over the first 24 h of culture at pH 6.0. The values are the mean of three replicates and their standard errors. Means with different letters are significantly different according to the Bonferroni Multiple Comparisons Test (*p* < 0.05).

The effect of different temperatures on AFB1 degradation by Actinomycetes was also studied. A general degradation of AFB1 was observed for all the strains at different temperatures. The optimal degradation temperature was 30 °C at pH 6.0 for all the strains. In addition, *Streptomyces* strains had no significant difference in AFB1 degradation between 25 and 30 °C (Figure 3). This may be due to the range of enzymes produced from these microorganisms or the compatibility of their enzymes to work at a wide range of temperatures. This result agrees with Guan *et al.*, (2008) [22] as they recorded that no significant difference was observed between 20 and 30 °C when *S. maltophilia* 35-3 was used to degrade AFB1. The results in this study showed that the optimal degradation temperature was at 30 °C and pH 6.0 for *R. erythropolis* ATCC 4277, while the optimal degradation temperature for *Streptomyces* strains was 30 °C and pH 5.0 (*p* < 0.05). In another work, the optimum temperature for *Flavobacterium aurantiacum* to break down AFB1 was 25 °C [19,25] whereas no significant differences were found between temperature 10 and 40 °C in terms of AFB1 degradation by using the cell-free extracts of *R. erythropolis* and *M. fluoranthenivorans* [19].

**Figure 4:**
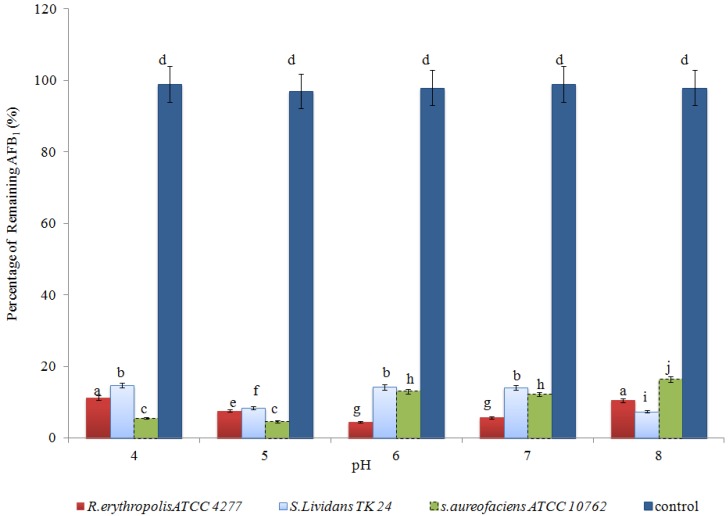
The effect of culture pH on AFB1 degradation at 30 °C over the first 24 h of incubation. Means with different letters are significantly different according to the Bonferroni Multiple Comparisons Test (*p* < 0.05).

The effect of initial culture pH on AFB1 degradation over the first 24 h incubation at 30 °C was examined (Figure 4). The results indicated that AFB1 was not stable in all the pHs used in this study. The most efficient degradation was around a natural pH with an optimum of degradation as no significant differences were found between pH 6.0 and pH 7.0 for *R. erythropolis.* In contrast, the optimum degradation for *Streptomyces* was achieved at pH 5*.* The initial pH did not have any significant effect on the amount of AFB1 in the control over a period of 24 h. This was in agreement with Ciegler *et al.*, (1966) [25] whose result showed the optimum pH for AFB1 degradation by *Flavobacterium aurantiacum* was at pH 6.75 and the rate of AFB1 degradation was decreased at pH 5 and 8, respectively. In another study, the optimum degradation of AFB1 by *Flavobacterium auranticum* was observed at pH 7, with some AFB1 degradation occurring at pH levels as low as 5 and as high as 8 [27]. However, the optimum degradation of extracellular enzymes of *Pleurotus ostreatus* was achieved at pH 4 and pH 5, and at 25 °C activities were found at pH 4 and pH 5 [11]. Meanwhile, Guan *et al.* (2008) [21] reported that the maximum AFB1 degradation by *S. maltophilia* 35-3 was at pH 8. However, identifying the optimum conditions and understanding the underlying key factors involved in the biodegradation process may assist in developing a model for use on an industrial Fermentation scale.

**Figure 5:**
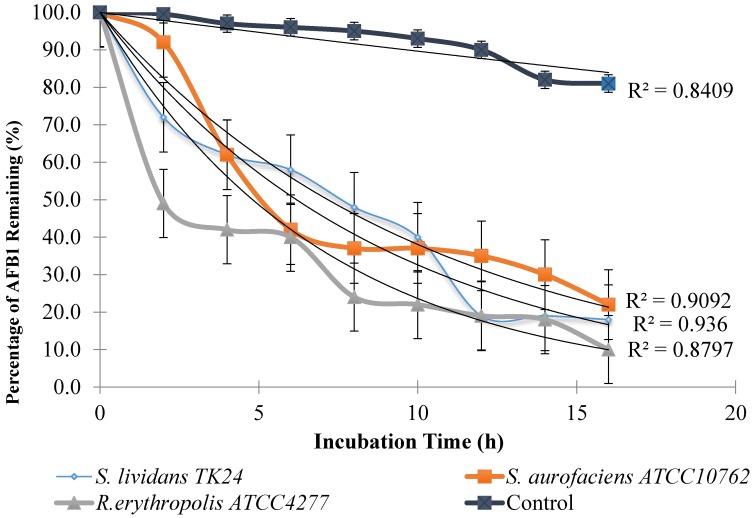
Kinetics of degradation of AFB1 over the first 16 h of incubation at pH 6.

The kinetics of degradation of AFB1 over the first 16 h incubation with Actinomycetes cultures at 30 °C and pH 6.0 is shown in Figure 5. There was no significant difference in the ability of each of the microorganisms to degrade AFB1 with increasing time. The rate of degradation by *R. erythropolis* ATCC 4277 was higher compared to the other two strains. After two and a half hours, 50% of AFB1 degraded, whereas 70% and 90% remained when AFB1 was exposed to *S. lividans* TK 24 and *S. aureofaciens* ATCC 10762 cultures, respectively. A significant difference (*p* < 0.05) was found between negative control and the treated samples. The control was stable over a period of 72 h. Comparable results were determined by Teniola *et al.* (2005) [19]. Their results indicate that 90% was degraded after only 4 h incubation with cell-free extract of *R. erythropolis*, whilst after 8 h AFB1 was not detectable*.* Furthermore, culture supernatant of *S. maltophilia* showed high degradation activity where about 78.8% was degraded after 72 h incubation [21].

### 2.3. Confirmation of AFB1 Degradation

Four techniques were used to confirm the degradation: TLC, Reverse phase HPLC, ion-trap ESIMS, and high resolution FTMS (HR-FTMS). The TLC result confirmed AFB1 degradation. The TLC showed clearly the cleavage of the lactone group. The amount of AFB1 fluorescence decreased within the first 24 h and disappeared after 72 h of incubation with Actinomycetes. However, it is known that opening the difuran ring will not affect the molecules’ fluorescence, while opening or abolishing the lactone ring does affect the fluorescence. Lee *et al.* (1981) [28] reported that AFB1 fluorescence is associated with the presence of an intact lactone ring as well as Motomura *et al.* (2003) [11] who reported that the decrease of AFB1 fluorescence indicated cleavage of the lactone ring. In this research, the Reversed phase HPLC also showed a decrease in the fluorescence intensity within the time, and a smaller peak was detected after 72 h of incubation with Actinomycetes. However, HPLC and TLC analysis did not reveal the formation of any breakdown products. Therefore, a more sensitive technique was required to confirm the degradation and to identify the breakdown products.

### 2.4. Breakdown Product Identification and AFB1 Degradation Hypothesis

**Figure 6:**
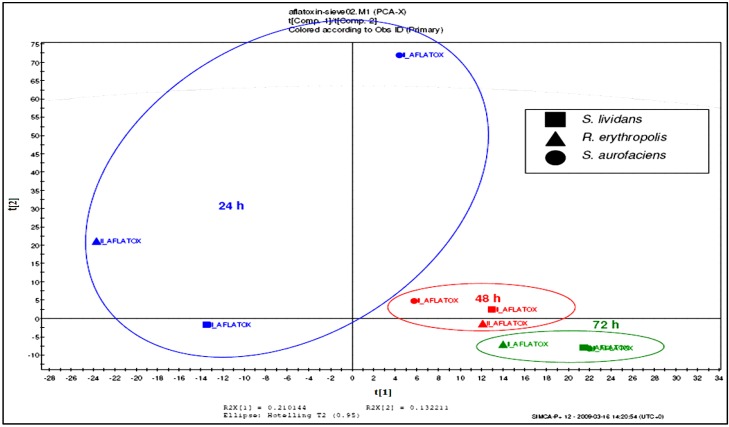
PCA and Multivariate data analysis of AFB1 by different Actinomycete strains at variable time frames.

The third aim of this study was to define a possible degradation mechanism of AFB1 by the microbial liquid cultures used in this research. The HPLC, ion-trap ESIMS, and HR-FTMS confirmed the cleavage of the lactone group, as the peak area designated for AFB1 was decreasing over time. Meanwhile, the LC-ESIMS indicated that another metabolite was being produced during AFB1 degradation. These results were consistent with the HR-FTMS results. The similarity and diversity of the samples at different incubation times were determined by principal component analysis (PCA) where similar samples were clustered (Figure 6). The result showed that the metabolites were clustered together according to each microorganism used. There was no significant difference in the ability of each of the microorganisms to degrade AFB1 with increasing time. However, it was also observed that the metabolites for *R. erythropolis* at 48 and 72 h clustered separately from the other two microorganisms. Greater emphasis was given to *R. erythropolis* as it was the most efficient in terms of AFB1.

**Figure 7:**
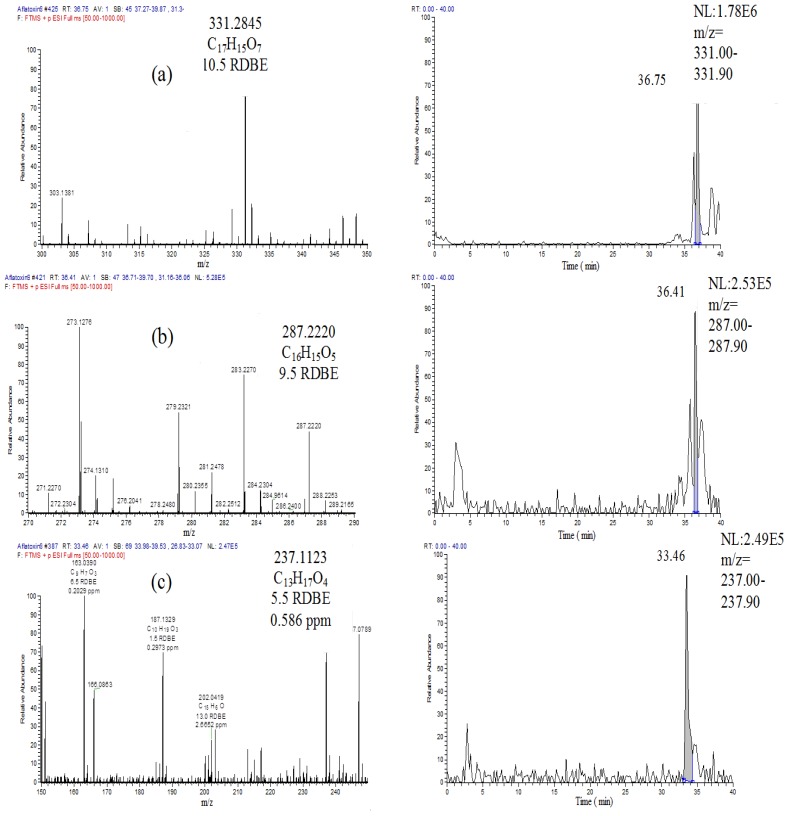
MS spectral data for [M + H]^+^ ion peaks at *m*/*z* 331.2845 (**a**), 287.2220 (**b**) and 237.1121 (**c**) with each of their extracted ion chromatogram after 72 h of incubation time with *R. erythropolis* culture.

**Figure 8:**
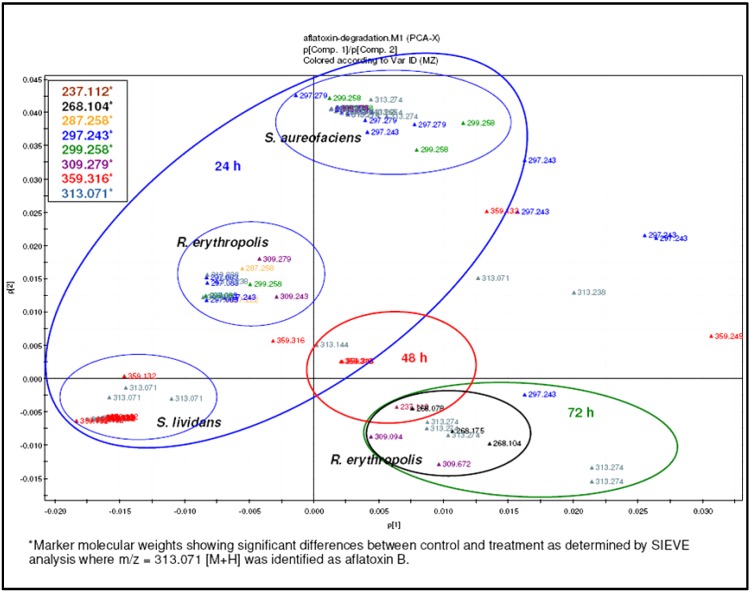
PCA analysis of AFB1 degradation by different Actinomycete strains showing the marker metabolites.

The high resolution FT mass spectroscopy LTQ-Orbitrap was utilised to confirm AFB1 degradation as well as to identify any of the metabolites produced during the biodegradation process. The mass spectral data from the Orbitrap were processed and analysed using the SIEVE software. The latter identified new mass ion peaks of other metabolites at nM concentrations. Treated samples were compared with the control at zero time. Significant ion peaks found in the treated samples were detected by SIEVE analysis including [M + H]^+^ ion peaks at *m*/*z* 331.2845, 287.2219 and 237.1121 (Figure 7) as well as the presence of other smaller molecules. These catabolic products were achieved during the *R. erythropolis* culture treatment. The formation of catabolic products, together with the presence or absence of AFB1, was evaluated by PCA analysis. It was observed that metabolites for *R. erythropolis* at 48 and 72 h clustered separately from the other two microorganisms. In addition, as generated by the SIEVE data which is shown in Figure 8, the [M + H]^+^ ion peak at *m*/*z* 237.1121 was being produced over a period of 72 h. It is clear that the concentration of this particular metabolite increased as AFB1 decreased.

SIEVE analysis and the correlation study identified which compounds were related to AFB1 degradation (Table 1). Positive and negative correlations were found between AFB1 and marker metabolites generated during the degradation process.

**Figure 9:**
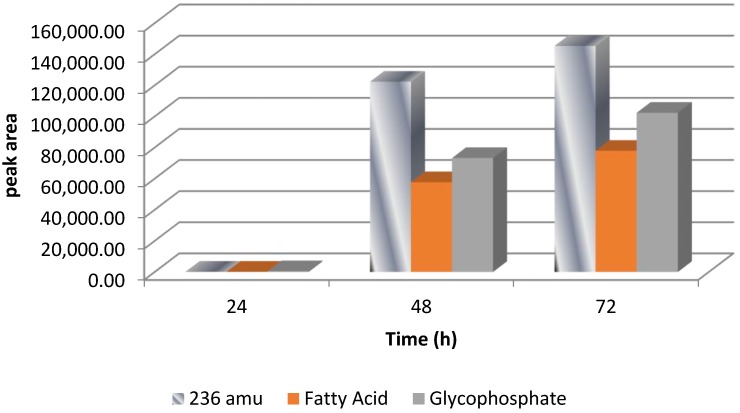
Summary of SIEVE-generated data for the formation of [M + H]^+^ ion peak at *m*/*z* 237.1121 along with fatty acid and glycophosphate content over a period of 72 h with *R. erythropolis* culture.

A highly negative correlation was found for the ion peak at *m*/*z* 237.1121 indicating a significant effect (*p* < 0.05) on AFB1 degradation (see Table 1), as the peak area of AFB1 was decreasing, and the total ion peak area for *m*/*z* 237.112 was increasing. AFB1 was degraded to a smaller metabolite of 236 amu with the suggested molecular formula of C_13_H_16_O_4_. Interestingly, over a period of 72 h incubation time, an increase of 236 amu metabolite was also associated with an increase in the fatty acid and glycophosphate compounds, as shown by the SIEVE-generated data, assisted with identified hits from the KEGG (Kyoto Encyclopedia of Genes and Genomes) pathway database (Figure 9). This also suggests the degradation of AFB1 to low molecular weight compounds prior to their plausible participation in the citrate cycle.

**Table 1:** Positive and negative correlation of marker metabolites generated during the degradation process of AFB1.

*m*/*z*	Peak area	Correlation	*p* value
331.2845	782442	−0.921	0.254
297.2428	2418033	−0.546	0.633
299.2584	117588	0.809	0.400
287.2219	115295	−0.645	0.263
269.2477	1488	0.916	0.554
237.1121	31685	−0.998	0.041

**Figure 10:**
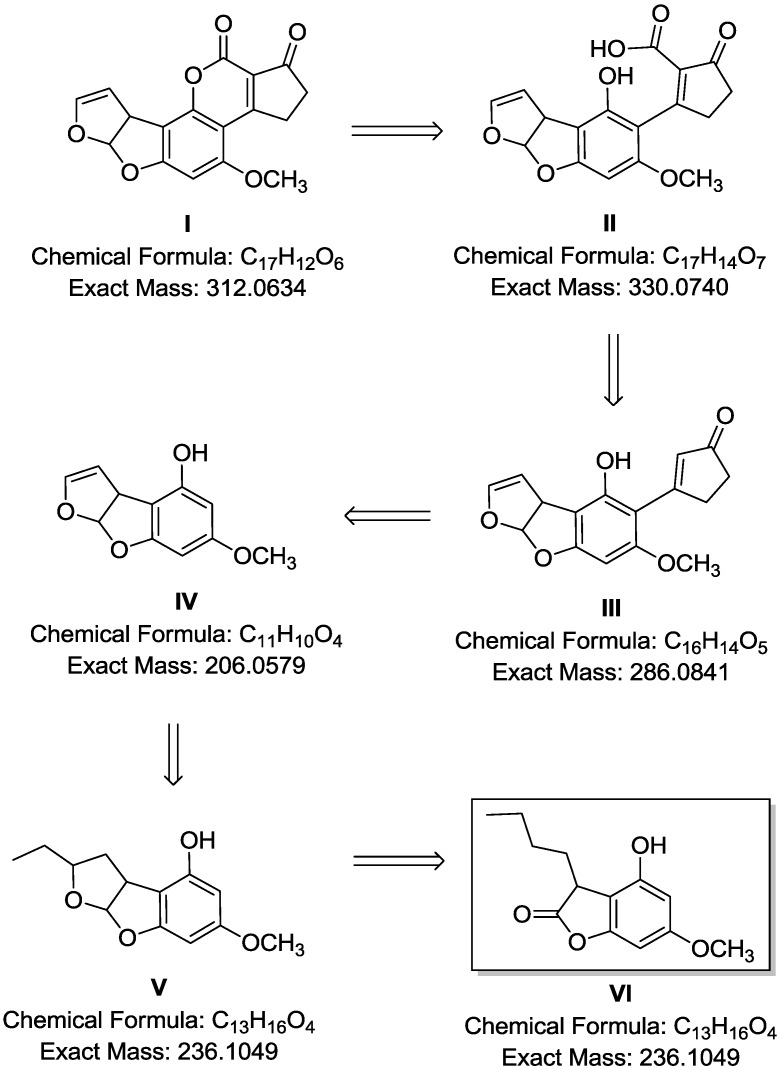
Hypothetical degradation mechanism for AFB1 by *R. erythropolis*.

Our hypothesis involves the degradation of AFB1 into another compound with chemical properties different from AFB1. Therefore, the HPLC run was set for 40 min to account for the formation of new metabolites. Furthermore, AFB1 degradation involves conversion of AFB1 into other analogues. High resolution FTMS results in this research were indicative of the formation of new metabolites along with the degradation of AFB1. The mass spectrum of the treated sample showed intense pseudomolecular ion peak values at *m*/*z* 331.0707, 287.2219, 237.1211 as well as that of at *m*/*z* 313.0707 attributable to residual AFB1. These ions were not present in the mass spectrum of either the reference sample of pure AFB1 or in the control samples. As a result, these metabolites were inferred as degradants achieved during the culture treatment. Consequently, the hypothesis for AFB1 degradation may involve the formation of the β-keto acid structure catalysed by enzymes produced by *R. erythropolis*, followed by hydrolysis of the lactone ring resulting in a metabolite with 330 amu (3.16-II). The hydrolysis was followed by decarboxylation of the open lactone ring yielding to 286 amu (3.16-III), which is known as AFD_1_. This involved the formation of 206 amu (3.16-IV), which is AFD_2_, where the difuran moiety was retained while the lactone carbonyl and cyclopentenone ring characteristic of an AFB1 molecule disappears. The enzymatic procedure involved cleavage of the unsaturated part of one difuran unit (3.16-V) yielding a furanolactone phenolic metabolite of 236 amu (3.16-VI) (Figure 10).

An early study indicated that AFB1 was most likely metabolised to degradation products with chemical properties different from those of AFB1, but they could not reveal the formation of any breakdown products through electron spray mass spectrometry. In another work, chemical inactivation of AFB1 and aflatoxin B_2_ (AFB_2_) in maize grain by means of 1N aqueous citric acid was reported [29,30]. Suttajit (2015) [31] suggested a complete degradation for AFB1 by chemical degradation; the hypothesis involved the use of high temperatures (220–340 °C) and ammonia. There are few studies on AFB1 biodegradation, and none of them show a complete degradation mechanism. Most of the proposed mechanisms in the biological system involved biotransformation of AFB1 into its derivatives.

Motomura *et al.* (2003) suggested that the specific enzyme cleaved the lactone ring of AFB1, although the degradation products of AFB1 were not investigated clearly; as the technique used was limited, they could not reveal any intermediate products [11]. In other research, a multi-enzyme from *Armillariella tabescense* was isolated and purified. These enzymes were capable of degrading AFB1 where the optimum activity was at 35 °C, pH 6.8. Their proposed pathway indicated the degradation of AFB1 by multi-enzymes: AFB1 was first transformed to its epoxide, followed by hydrolysis of the epoxide to give a dihydrol. Then, the difuran ring would open in the subsequent hydrolysis step [6]. However, the conversion of AFB1 to its epoxide is unlikely as it is more toxic and carcinogenic. Alberts *et al.* (2006) [32] and Teniola *et al.* (2005) [19] suggested that *Rhodococcus* could use a cascade of enzyme reactions to degrade AFB1 without suggesting any hypothesis for the degradation. Furthermore, Lapalikar *et al.* (2012) [33] identified and characterised enzymes from *R. erythropolis* that could degrade AFB1 into smaller compounds.

In this study, the interesting results were detected by HR-FTMS analysis, suggesting that AFB1 degradation is associated with the accumulation of intermediates of fatty acid metabolism, and glycolysis. Various enzymes were identified from *R. erythropolis* involving catabolic pathways of aromatic compounds such as polychlorinated biphenyls enzymes, which included ring cleavage, biphenyl dioxygenases, dihydrodiol dehydrogenase and hydrolases [10]. Furthermore, all the central pathways for aromatic degradation ended with the citrate cycle [10]. AFB1 is a polyaromatic compound, and the degradation may occur in the same pathway as mentioned for the aromatic compound. However, more investigation is required to identify the enzyme or enzyme systems responsible for the biodegradation.

## 3. Experimental Section

### 3.1. Microbial Strains and Cultivation

Biological degradation of AFB1 using three different strains of Actinomycetes was investigated in this study. The *R. erythropolis* strain was selected based on published data [19] while the Streptomyces strains were tested against AFB1 for the first time. Strains of (*Streptomyces lividans* TK 24 and *Streptomyces aureofaciens* ATCC10762) used in this study were obtained from the Institute of Pharmacy and Biomedical Sciences (SIPBS) collection at the University of Strathclyde, Glasgow, UK. *R. erythropolis* ATCC 4277 was purchased from (ATCC LGC Standards, Bury, UK). The strains were cultivated in Difco ISP medium No.1 comprised of 0.5% (*w*/*v*) pancreatic digest of casein and 0.3% (*w*/*v*) yeast extract (Sigma Aldrich, Dorset, UK). The cultures were stored at −80 °C in 20% glycerol until use. Prior to use, the strains were inoculated into 250 mL of Difco ISP medium No. 1 and incubated at 30 °C for 48 h and pH 6.0 at 200 rpm on a shaker (New Brunswick Scientific, Edison, NJ, USA).

### 3.2. AFB1 Degradation by Actinomycetes in Liquid Culture

0.2 mL of a 100 µg/mL AFB_1_ dissolved in methanol (Sigma Aldrich, Dorset, UK.) was added to 2 mL Eppendorf tubes. The methanol evaporated before adding the culture. Then 50 µL of 48 h pre inoculums were inoculated into 0.75 mL of Difco ISP medium No. l, adding to 2 mL sterile screw-capped Eppendorf tubes to a final concentration of 20 µg/mL. The cultures were incubated at 30 °C for 24, 48, and 72 hours. Microbial growth was monitored by optical density observations of the cell suspension at 600 nm. The cells were removed by centrifugation (JuanBR4i Multifunction, ThermoScientific, Porton Down, UK) at 11,000 rpm for 15 min at 4ºC. AFB1 was quantified by using reversed phase HPLC as described below. Sterile medium supplement with AFB1 with a final concentration of 20 µg/mL was used as control.

### 3.3. Optimization of the Degradation Conditions

The degradation of AFB1 at different temperatures, pH and incubation times was investigated to determine the optimum condition for degradation by the different strains. The chosen temperatures were 25, 30, 35, and 40 °C over a period of 24 h at pH 6. The pH values used in this experiment were 4, 5, 6, 7, and 8. The cultures were incubated in the dark for 24 h at 30 °C. The pH was adjusted by using 1 N HCI or 1N NaOH as required. The control was used in this study (media+AFB1 20 µg/mL) and AFB1 was quantified by using reversed phase HPLC as described below. All experiments were performed in triplicates.

### 3.4. Quantification of AFB1 and Its Degradants

Extracting AFB1 from the medium was not necessary as one of the study aims was to purify and identify the degradants. Four methods were used in this study to confirm AFB1 degradation: Thin layer chromatography (TLC) (Sigma Aldrich, Dorset, Gillingham, UK), High-performance liquid chromatography (HPLC) (Gilson instrument, Luton, Bedfordshire, UK), Liquid chromatography-mass spectrometry (LCMS) (ThermoFinnigan, Bremen, Germany) and high resolution Fourier transform and orbitrap mass spectrometry (Orbitrap FTMS) (ThermoFinnigan, Bremen, Germany).

#### 3.4.1. TLC

TLC analysis was done on silica gel (Si_60_) plates (Sigma Aldrich, Dorset, Gillingham, UK). The plates were developed using chloroform: acetone (9:1, *v*/*v*) as solvent system and monitored under UV at 365 nm.

#### 3.4.2. Reversed phase HPLC

Reversed phase HPLC (Gilson instrument) analysis was performed through a RP-C18 (5 μm, 150 × 4.6 mm ID) column (ACE, Hichrom limited, West Berkshire, UK) attached to a C6-phenyl 4.0 × 3.0 mm ID guard column (Phenomenex, Macclesfield, Cheshire, UK). Elution was achieved at a flow rate of 1 mL/min with acetonitrile: methanol: water (1:1:2, *v*/*v*/*v*) as the mobile phase. Injection volume was 20 µL. The sample temperature was controlled at 40 °C by using column heater model 7971 (Jones chromatography, Mid Glamorgan, UK). A photo-diode array detector (PDA) was used to monitor the presence of AFB1 by UV detection (Gilson, Luton, Bedfordshire, UK) at a wavelength of 365 nm. The data were collected and processed with Gilson unipoint LC system software (Gilson, Luton, Bedfordshire, UK). A control was used in this study (media + AFB1 20 µg/mL). The percentage of remaining AFB1 was calculated using the following formula:

$$\text{Percentage of AFB}1\text{ Remaining}=\left(\frac{\text{AFB}1\text{ peak area in the treatment}}{\text{AFB}1\text{ peak area in control}}\right)\times 100$$

#### 3.4.3. LCMS

The LCMS procedure was used to further confirm that AFB1 degradation was performed on a HPLC-PDA Agilent 1100 system coupled with the LCQ Deca XP (ThermoFinnigan, Bremen, Germany) ion trap instrument equipped with an electrospray ionization (ESI) source. Separation was accomplished on a reversed phase ACE 5 C18-300 5 μm, 300 Å, 30 × 4.6 ID mm column (Hichrom Limited, Reading, UK). The mobile phase consisted of acetonitrile: methanol: water (1:1:2, *v*/*v*/*v*) with 0.01 M of ammonium formate buffer at a flow rate of 0.4 mL/min. UV and mass spectral data were recorded for 5 min. ESI mass spectra ranging from *m*/*z* 50 to 1000 amu were taken in the positive-ion mode. The pseudo-molecular ion peak at *m*/*z* 313 [M + H]^+^ for AFB1, was monitored through its MS^2^ data. The MS detector (ThermoFinnigan, Bremen, Germany) was set at a vaporizer temperature of 220 °C; a sheath gas flow rate of 50 arbitrary units; auxiliary gas flow rate of 10 arbitrary units; source voltage was at 5 kV; capillary voltage at 15 V; and the tube lens offset at 30 V. Two scan events were run; the first was the full scan range from *m*/*z* 50 to 1000 in positive mode and the second run was an MS^2^ fragmentation for *m*/*z* 313 set at the range of 85–500 amu at the same mode. A control was used in this study (media + AFB1 20 µg/mL), and the mass spectral data were processed with Xcalibur 2.0 (ThermoFinnigan, Bremen, Germany).

#### 3.4.4. LTQ-Orbitrap

The high resolution Fourier transform mass spectroscopy technique was achieved with the LTQ-Orbitrap to identify the metabolomes produced during the biodegradation process. The LTQ-Orbitrap (ThermoFinnigan, Bremen, Germany) instrument equipped with an ESI source was coupled to a Surveyor HPLC-PDA (ThermoFinnigan, Bremen, Germany) system. The HPLC conditions were identical to those employed on the LCQ Deca XP instrument. However, a gradient run was used, commencing with 5% acetonitrile for 5 min and increased to 100% after 35 min with an additional 5 min at 100% acetonitrile. Spectral data were recorded for the entire 40 min. AFB1 was detected and eluted at 12.5 min. The MS detector was set at a vaporizer temperature of 220 °C; a sheath gas flow rate of 30 arbitrary units; auxiliary gas flow rate of 10 arbitrary units; the source voltage was at 4 kV; the capillary voltage was at 35.5 V, the tube lens at 30 V; and the capillary voltage was at 21 V. The data were processed by Xcalibur 2.0 while metabolomic studies were done using SIEVE 1.2 (ThermoFinnigan, Bremen, Germany), differential analysis software to assist peak monitoring at lower thresholds. The SIEVE software was connected with the online library ChemSpider to detect aflatoxin analogues and other smaller molecule degradants. The same set of data generated by SIEVE was exported to SIMCA P + 12 (Umetrics, Umeå, Sweden) for principal component analysis to determine any similarity and differences in metabolome formation during the degradation process by the different strains at different incubation times. Two controls were used (media + AFB1 20 ia + ols were used d at 12.5 min. The AFB1 was used as a blank control to enable the exclusion of background mass ion peaks belonging to the culture media.

## 4. Statistical Analysis

Data on the effect of the pH, temperature and time on the response variable AFB1 concentration were statistically evaluated using one and two-way ANOVA. The results were considered significant when *p* values were *p* < 0.05. Tukey’s multiple comparison tests were done when significant difference was encountered. In addition, multivariate analysis using SIMCA was used to analyze the data. The purpose of using SIMCA is to determine the similarity of a group of samples according to their principle component where each group is described as a cluster. Furthermore, a correlation between AFB1 degradation and certain metabolites was analysed with Minitab software.

## 5. Conclusions

In this study, biological degradation of AFB1 by 3 Actinomycete species, (*Rhodococcus erythropolis* ATCC 4277, *Streptomyces lividans* TK 24, and *S. aureofaciens* ATCC 10762) was examined in liquid cultures. The degradation of AFB1 by these cultures was achieved without pre-exposing the cultures to the toxin. Our study extended the range of microorganisms capable of degrading AFB1. No significant difference was found between the three cultures in terms of ability to degrade AFB1 over a period of 72 h. The degradation was temperature, time and pH dependent. However, each microorganism has a different way of degrading AFB1. The metabolites produced during AFB1 degradation by *R. erythropolis* were significantly different from those produced during degradation by the other two microorganisms. The research gained new insight into the biodegradation pathway by *Rhodococcus* and elucidated the factors that influence metabolite degradation. TLC assay confirmed the cleavage of the lactone group by *Rhodococcus.* A hypothetical degradation mechanism for AFB1 by *R. erythropolis* was proposed in this study based on our result and linked to the published work of others. The degradation of AFB1 was associated with the increase of fatty acid and glycophosphate metabolites. Our findings provide valuable information that can be used to devolve a model for industrial fermentation for food processing or bioenergy production. More work is still required to gain a better understanding of AFB1 degradation by these microorganisms. Further investigation of toxicity and enzyme identification and purification involved in toxin biodegradation by Actinomycetes is undergoing research.
